# Collectanea

**Published:** 1829-05

**Authors:** 


### COLLECTANEA.

Floriferis ut apes in saltibus omnia libant,

Omnia nos, itidem, depascimur aurea dicta.

### PHYSIOLOGY.

*Experiments relating to the Reproduction of the Crystalline Lens.* By MM. Cocteau and Leroy d'Etoille. (*Magendie's Journal de Physiologie.*)

The means which nature employs is not often used to assist the loss which the eye receives by the extraction of the crystalline lens. Authors, for the most part, are silent on this subject; and those who have fixed their attention to it positively deny the reproduction of the lens. Thus Haller, in his "Elementa Physiologiæ," says that the lens, being a solid, cannot, like the humors, regenerate itself: "Lens cristallina fabricam sibi propriam solidam habeat neque humoribus debet accenseri, neque amissa renascitur."

The dissection of an eye deprived of its lens, says M. Tartra, presents remarkable appearances: at first the depression of the vitreous body, which corresponds to the posterior segment of the lens, is not replaced by any new body; the vitreous humor fills the space which it occupies; the capsule of the lens does not exist, it is removed by absorption. (*Thèse présentée au Concours de* 1811.) Already TENON has announced this fact: "Cristallini fossula ab humore vitreo in ejus locum secedente repletur." Wishing to know if the observations or the assertions of the authors cited are correct, we have practised experiments on different animals, and have obtained results quite different to those which they have announced. We will not examine if this difference is because the researches have been made on eyes operated on in a perfect state, whilst in the other they have been made on eyes operated on account of cataract; we will relate only the results of our experiments; their accuracy may easily be verified by similar experiments.

MM. COCTEAU and LEROY D'ETOILLE performed six experiments, by extracting the lens; and in all the cases they found, in one or the other eye, traces of formation of a new lens. We give their last experiment as an example.

"On the 6th of June, 1825, we performed the operation of cataract on the eyes of two rabbits. After the lapse of a month, both could see very well; but through some accident one was killed, without our having an opportunity of examining the eyes. The other was kept till the 18th of November, (i. e. more than six months,) when the examination of the eye was made in the presence of M. FODERA. The crystalline capsules were perfectly transparent, no cicatrix whatever could be observed; they contained lenses of a moderate size, and of as firm a consistence as those that we extracted. In order to acquire more certainty as to their nature, we plunged them into boiling water, and saw them immediately become opaque, hard, and friable, like the primitive lenses; merely the arrangement into bright lamellæ was only evident in the eccentric lamellæ.

From the experiments which we have just mentioned, it evidently results that the lens may be reproduced. But this fact, not known, or forgotten up to this day, gives rise to other questions. That the lens is reproduced, is it necessary that the eye should be in a perfect state to the moment of extraction? The lens being removed on account of cataract, is it replaced by another? The persons who, after the operation for cataract, could see without the aid of convex glasses, do they owe this faculty to the formation of a new lens? Will the lens continue to be classed among the living solids, or can this property of reproduction be likened to the other humors of the eye?—We will content ourselves at present with proposing the questions, and will defer answering them till we have gathered sufficient facts to enable us to form an opinion.

---

### PATHOLOGY.

*Case of a Man who fancied that he had a Serpent in his Belly, and upon whom the Operation of Gastrotomy was pretended to be performed with success.* (Condensed from *La Clinique.*)

The patient was a young countryman, a labourer in the fields, who fancied that he had swallowed a small serpent in a glass of muddy water. This alarming accident had occurred five years before he presented himself to M. MAURY, at the hospital of St. Louis. The animal had grown to an enormous size, in the imagination of the unfortunate patient, and there was scarcely any part of the body upon which its visits were not occasionally inflicted: its most common residence, however, was the abdomen. Labouring under such an hallucination, the mental distress of the patient may be imagined. He was firmly convinced that nothing but an operation could relieve him from his tormentor; and, as it was evidently useless to reason with him upon the subject, the following *ruse de chirurgien* was practised.

To render the illusion complete, a large fold of the abdominal integuments was drawn up, and a bistoury was passed through. A living adder was secretly introduced into the wound, like a seton. The patient was then allowed to grasp the bleeding head of the animal, and to assist the surgeon in removing it from his body; which was, of course, happily effected. He felt the greatest joy at his deliverance, and was fully sensible the next day of the wonderful diminution of the size of his belly. The dreadful sensations he had experienced for five long years, and the hissing of his tormentor, no longer troubled him. He was completely well in a few days, and has since had no complaint.

One circumstance nearly renewed all his anxiety. He feared that some eggs of the serpent might be left behind; but this apprehension was removed by the adroit assurance of the surgeon that his late visitor was a male.

---

*Diseases of the Heart caused by Onanism.*—Dr. KRIMER, of Aach, has lately published an interesting paper on this subject. Our own experience has furnished us with several opportunities of seeing cases of the kind he describes; and, as the subject has not hitherto been particularly discussed, we shall give the leading points of his communication.

Dr. K. is of opinion that diseases of the heart, which have increased so much within the last twenty years, do not always depend upon organic alteration, but are very frequently produced by the baneful and lamentably frequent practice of the vice of onanism. Headachs, great anxiety, palpitations, faintness, an oppression and unusual sensibility in the epigastric region, are the first symptoms produced. They increase in severity in proportion as the subject gives way to the gratification of his unnatural propensity, and quickly diminish, or cease altogether, if he abandons it. To support his opinions, M. K. states many cases. He enumerates the following symptoms as pathognomonic of such affections of the heart; by an attention to which, the practitioner will be enabled to distinguish the train of symptoms from other diseases which are not unfrequently suspected.

1. The hair loses its natural brilliancy, is remarkably dry, and frequently splits at the extremities. It falls off easily and in large quantities, especially from the fore part of the head. In persons affected with consumption, or organic disease of the heart, the hairs appear well nourished, and rarely fall off.

2. The eyes are dull, downcast, frequently full of tears, and without expression, and deeply sunken in their orbits. The edges of the eyelids are reddish, and surrounded by a bluish tint. In phthisical patients, and those with organic disease of the heart, the eyes are brilliant, and always preserve their natural expression and vivacity. In young females, at the approach of menstruation, a blue circle is commonly observed around the eyes, but here also their brilliancy is undiminished.

3. The patient appears very timid, and unwilling to look other people in the face.

4. Periodical headach is common, extending from the occiput towards the forehead.

5. The power of sight is diminished; the appetite is lost, the tongue is usually loaded. A slight cough, short and difficult respiration, are generally present; but still the patient can draw a deep inspiration.

6. Pains in the stomach, with weight and pressure in the epigastric region. Patients with organic diseases of the heart have occasionally these symptoms, but in such cases they are not accompanied by those above enumerated.

7. A general feeling of lassitude and feebleness of the limbs, with pains in the lower part of the back. We would add, also, that pain and throbbing of the testicles, with uneasy sensations shooting up the spermatic cord, are frequently complained of.

8. The perspiration has a dull and sweetish odour, similar to that of infants at the breast.

9. If the vice of onanism be touched upon in conversation, the agitation and embarrassment of the patient invariably betray him.

10. If the practice be continued, the mind is at length enfeebled, the patient is incapable of mental or bodily exertion, and sinks into a state of somnolency.—*Hufeland's Journal.*

---

*Abscess in the Cavity of the Meninges, cured by the Application of the Trephine.* By M. Roux.—A boy, aged fifteen, had, for more than four years, a small fistulous opening in the left parietal region, in consequence of the opening of an abscess of the scalp caused by a blow. M. Roux, having been consulted, thought that there existed caries of the internal table, and that coma, which occurred when the pus did not flow freely, was owing to the accumulation of pus from this caries. He applied the crown of a trephine upon the fistula, with the intention of facilitating the passage of the portions of caries, or necrosed bone: he then found that caries did not exist; the fistula penetrated the dura mater. This membrane was incised, and he found that there existed an abscess in the cavity of the meninges, which compressed the brain. When the pus was evacuated, he perceived a considerable depression on the middle of the left lobe; but this depression gradually diminished, and finally disappeared. The wound made by the trephine gradually healed, and the patient was cured.—*Journal général de Médecine.*

---

*Absence of the Menses in a Woman in whom the other Signs of Puberty existed; Obstruction of the Extremities of the Fallopian Tubes, with Enlargement of the Uterus, which contained a membranous Bag full of Pus.* By A. Reynaud.

A native of Flanders, who, though twenty-one years old, had never menstruated, was admitted a patient of the Hôpital la Charité, with disease of the spine, excepting which and small-pox she had never been ill. At thirteen she had the appearance of a woman, after which, though tall and well made, she grew but little. She never had the precursory signs of incipient menstruation, nor was she subsequently, at any period of the month, similarly affected. At the age of eighteen, she complained of a dull fixed pain in the lumbar region; yet the influence of habit enabled her still to follow her laborious occupations; but, the pain gradually increasing, she was soon almost, and ultimately quite, a cripple. Two months before her admission into the hospital, an abscess formed at the anterior and superior spinous process of the left ilium. When it was first examined at the hospital, the skin was of a natural colour; the tumor, of the size of a large walnut, fluctuated and disappeared by pressure. As yet there were no signs of fever. The usual treatment of psoas abscess was instituted; but the disease became daily worse, and the patient at length died, worn out by constant suppuration and pain.

After death, one of the vertebræ was found to be nearly destroyed; the femur, ilium, &c. were also much diseased.

The uterus was large enough to be mistaken for that of a woman about six weeks pregnant; exhibited very evident fluctuation, but no fluid escaped through the neck, although this part, more than commonly capacious, was pervious, but partly filled by a whitish, glairy, cohesive substance. An incision being cautiously made through the parietes of the womb, its cavity was discovered to be enlarged and completely occupied by a false membrane, which closed up the orifice of the neck and those of the fallopian tubes. This sac adhered rather intimately to the walls of the uterus, from which it was easily separated, though not united to them by cellular membrane. This sort of cyst contained about three or four ounces of a yellow, grayish pus. Its internal surface resembled a mucous membrane without villi. A black substance, like the uvea, was deposited irregularly between this adventitious membrane and the uterus, whence it derived its mottled appearance. It was corroded internally in several parts, but not quite through; yet two of these ulcerations were extensive, deep, and bounded by the substance of the uterus, itself become soft and irregular. These ulcerations were situated posteriorly, and therefore at a distance from the spot where the neck of the uterus and fallopian tubes usually open.

No trace whatever of the uterine orifice of the fallopian tubes could be found. An incision was made along the course of these tubes, and a hog's bristle was attempted to be thence passed into the uterus, but this was found to be impracticable, since they became impervious as they approached the cavity of that organ, whence they were intercepted by a thin layer of uterine substance, and their diameter was in general much contracted. The caliber of the fallopian tubes was obliterated towards the ovaries. These bodies were more than an inch long, and several small cicatrices were observable upon their surface. Both contained in their centre a corpus luteum, of a brown red colour; and several small fibrous bags in a wrinkled and collapsed state, but admitting of considerable dilatation. Many small oval bodies, as large as hempseed, hollow and of a fibrous texture, somewhat resembling small eggs, were found in the course of the fallopian tubes, and within the folds of the broad ligaments of the uterus.—*Journal Hebdomadaire.*

---

*Obliteration of the left Iliac Vein; Venous Circulation continued collaterally, &c.* By M. Reynaud.

In the veins, as well as in the arteries, the course of the blood is not necessarily interrupted by the obstruction of a large venous trunk, since it may be kept up by collateral anastomosis, as well as by other means. This is accomplished exactly in the same manner as in the arterial system: at first a great many minute veins augment in size, and assist in conveying the blood from the parts below to those above the obstruction; beyond which some of the veins gradually assume their proper volume, while a few of them retain their newly acquired caliber. Although this state of the venous circulation has not been frequently, it has been correctly described; yet such is the importance of the fact in a practical as well as physiological point of view, and especially in the diagnosis of some cases of dropsy, that the following details of a case, which was recently treated at the Hôpital la Charité, appears to be worthy of record.

A woman, aged sixty-one, who had been for some time under the care of the physician, came under that of MM. Boyer and Roux, on 22d July, for disease of the right haunch. Five months before she began to feel pain in that part; some weeks after it extended to the knee, and rendered walking difficult. She affirmed that she had never fallen upon her haunch, or in any way bruised it. At this period the right haunch was swollen, and the skin replete with varicose veins. This thigh was about two inches shorter than the other; the great trochanter was observed to be nearer the spine of the ilium than is usual.

The nature of the disease was immediately recognized, and its fatal termination anticipated. The attention of the surgeon was particularly attracted by a very large vein, which, from one of the crural veins, extended across the abdomen, making many irregular turns till it reached the umbilicus, and then it returned back to join the opposite crural vein. At its origin it was single, and afterwards divided into two or three branches, which, soon uniting again, terminated in a single trunk. When the patient was in the erect position, this vein was as large as the little finger. It appears that the patient did not notice the unusual size of this vein till after she had been affected with pain in the haunch and difficulty of walking.

MM. Boyer and Roux, being persuaded that this was a case in which, owing to obliteration of a large vessel, the circulation was kept up collaterally, and that nearly all the blood of the left was conveyed into the right crural vein, they endeavoured to ascertain the precise direction in which the blood flowed, and found that it circulated from left to right. Though there was little doubt as to the obliteration, more or less complete, of the left iliac vein, yet its precise date was not known. Though the patient affirmed that it had but recently existed, yet she related that, between the age of ten and seventeen, her left inferior extremity became œdematous, and the least exercise made her foot swell so much that she was obliged to go slipshod. The swelling subsided by repose. She married at twenty-one, and, on her becoming pregnant, both her legs became œdematous, but the left considerably more so than the right. After her confinement, the swelling of the right leg disappeared, while that of the left continued as before. At this time she was subject to palpitation, to colic, and a slight cough; and from time to time the submaxillary glands were enlarged, though her health was not in other respects bad. At a later period she took a voyage to Martinique, where she resided several years.

During her abode in the hospital, and long before, she was extremely thin, the pain in the haunch unremitting, and latterly she was reduced to a mere skeleton. The feet were both œdematous, but the left more so than the right. The appetite failed; diarrhœa supervened; the gums and tongue were covered with aphthæ; deglutition became difficult; she had frequent nausea and pain in the epigastrium. At this time her debility was extreme, pulse small and fluttering, and she died on the 4th of August.

*Inspection of the body.*— The iliac vein is entirely obliterated from just above the hypogastric to where the superficial vein of the abdomen joins the crural, about the space of four inches. Throughout its whole course it is surrounded with a cancerous production, from which it is difficult to distinguish it at first sight; but, by attentive dissection, it is found to traverse this mass, and, after appearing a mere cord, solid and compact, again becomes pervious towards the crural arch. The hypogastric vein and its branches were in the same manner obliterated. At the part where the vena saphena empties itself into the crural, a large venous trunk arises from the latter, which is discovered, both by its course and origin, to be the superficial vein of the abdomen. At first taking an oblique direction upwards and inwards, it rises as high as the umbilicus, subdivides into three large branches, which, assuming the form of an arch, descend towards the crural of the other side, after again becoming a single trunk. Near its origin likewise arises another branch, less voluminous, which, after transversely crossing the hypogastric region, terminates in the right crural vein, at a part corresponding to its commencement in the left. Thus it became sufficiently manifest that the blood, returning from the left inferior limb, had ceased to pass by the iliac vein of that side, but, on the contrary, circulated through the right iliac by means of the collateral branches. The crural vein, where the superficial one of the abdomen enters it, is unusually large; and the caliber of the corresponding iliac equally augmented. (*Ibid.*)

---

*Case of Inflammation of the Umbilical Vein, with Infantile Erysipelas.* By Robert Lee, m.d. Physician to the British Lying-in Hospital.

An infant, four days after birth, was attacked with erysipelatous inflammation of both forearms, and severe febrile symptoms. Two days after the first appearance of redness and swelling of the integuments of the arms, a similar affection was perceived in those of the hypogastrium, genital organs, and upper part of the thighs. The child died on the twelfth day subsequent to birth, and was examined on the 18th November, 1828, two days after death.

The cellular tissue of the affected parts was highly vascular, and in the inguinal regions infiltrated with serum. On opening the abdomen, the peritoneum covering the different viscera was found in a healthy condition; but the umbilical vein, from the navel to the liver, was preternaturally indurated and distended. On laying it open, a yellow-coloured purulent fluid escaped, and the whole of its interior surface was found lined with a layer of closely adhering lymph: this coating of lymph extended into the principal branches of the umbilical vein, proceeding to the liver, and along the ductus venosus, as far as the vena cava. The umbilical vein and ductus venosus remained pervious, and there was no morbid appearance in the vena cava above or below its entrance. The coats of the umbilical were much more thickened than they are usually found to be at the same period after birth.

Morbid appearances of the umbilical vein in young children, similar to those which I witnessed in the foregoing case, have not been described by any pathologist in this country, as far as I have been able to ascertain. In the writings of Gaithshore, Bromfield, and Underwood, we find little, indeed, that is satisfactory, respecting either the nature of infantile erysipelas or the alterations of structure which accompany it. That inflammation and suppuration of the umbilical vein and its branches not unfrequently occasion fatal erysipelas, or death, without any inflammation of the surface, in new-born children, I am disposed to infer, not from the preceding instance alone, but from the observations of several continental writers on the subject, and from the acknowledged pernicious effects of purulent matter when secreted within the veins of the adult.

Professor Osiander found the lungs inflamed, and the umbilical vein, from the navel to the liver, filled with a yellow fluid, in a child who died of erysipelas a short period after birth. In the body of a child, seven days old, examined by Meckel, the umbilical vein was found inflamed, and its inner membrane covered with a layer of pus, and perforated with small ulcerations. In another child, attacked soon after birth with vomiting, colic, diarrhœa, jaundice, and fever, he found the peritoneum inflamed, and a puriform effusion in the abdominal cavity. The branches of the vena portæ, and those of the umbilical vein, were swollen, and their coats much thickened.

M. Breschet has repeatedly observed this inflammation of the umbilical vein and its branches in the bodies of children who had died a few days subsequent to birth; and he is disposed to consider this phlebitis as the sole cause of death in many of these cases.

---

### PRACTICAL MEDICINE.

*The Efficacy of Tannin in Suppressing Uterine Hemorrhage.* By Dr. Porta. (*Annali Universali di Medicina.*)

Case I.—In the first case in which the tannin was given, the lady had been subject to hemorrhage, more or less, during a year; and the result was great emaciation and debility. Various remedies had been employed without success: a half-drachm of the dried leaves of the black muscadel vine was prescribed, mixed in a small quantity of water. The first dose was taken on an empty stomach, and the same quantity was repeated in about an hour after having taken food. This quantity produced no derangement of the stomach, and its action was so prompt that on the same day the hemorrhage ceased, and did not again return.

The success of this case induced Dr. Porta to try the remedy in some others.

Case II.—A lady, aged thirty-four, of a sanguineous temperament, of a robust constitution, who had regularly menstruated, and who was the mother of four children, had been subject to hemorrhage for a month, but which was so slight that she did not deem it necessary to seek medical aid. After having taken a journey, the hemorrhage increased very much, with violent pains in the hypogastric and lumbar regions. The duration and the urgency of the case, the hardness and the fulness of the pulse, determined me (says Dr. P.) to take some blood from the arm; and this bleeding I repeated at the end of two days, which succeeded in procuring an alleviation of the hypogastric pains, and in moderating the hemorrhage. I ordered the lady to take small doses of ipecacuanha and nitre, and to remain quiet. Finding, after several days, that the hemorrhage continued, notwithstanding the removal of the symptoms of irritation, I ordered a half-drachm of the leaves of the black vine every three hours; which was continued until three ounces had been taken, without any perceptible benefit. I was led to consider that the only useful part of the powder was the tannin which it contained, and therefore prepared it from the leaves in the manner recommended by Proust. Having thus procured tolerably pure tannin, I ordered the patient to take two grains of it made into a pill, with a little syrup, every three hours during the day. It appeared to agree very well with the stomach; and at the end of three days the hemorrhage ceased; and the lady has been since very well.

Case III.—Quaroni Angiola, aged about forty, was seized, on her recovery from a bilious fever, with severe uterine hemorrhage; and thinking this was only an irregular appearance of the menses, which had not altogether ceased, she did not at first notice it much. As the discharge, however, continued for some weeks, and the patient began to lose her appetite and strength, she applied to Dr. Porta for relief. Recourse was had immediately to the tannin, in doses of two grains every two hours; and in two days the bleeding entirely ceased.

Case IV.—Muffi Rosa, thirty-two years of age, of a delicate constitution, mother of an infant which she was then nourishing, was seized, in the fourth month of lactation, with copious uterine hemorrhage, which continuing for several days, she became alarmed, and applied to Dr. Porta. At the time he saw her, the hemorrhage had existed for twenty days, with considerable fever and pains in the loins; the abdomen was soft, but the hypogastrium slightly tumid. The tannin was prescribed in pills, as in the preceding cases; eight of which served to stop the hemorrhage entirely, and to remove all the symptoms of debility consequent on it.

Case V.—Aguzzi Teresa, aged thirty-eight, of a sanguineous, irritable temperament, had had uterine hemorrhage for three weeks, when I was called to her. On pressing on the hypogastrium, I remarked that it produced pain, and that the pain extended towards the loins. She had irregular accessions of fever, and the pulse was frequent; but there was no notable tumor in the hypogastrium. I considered that the tannin might be prescribed with effect, as in the former cases, and ordered three grains to be taken every three hours. About two scruples were taken in the course of about ten days, at which time the hemorrhage had entirely ceased.

Dr. Porta states that these are only a few of the numerous cases in which he has employed the tannin with success; and, during three years, he has only met with two cases in which it failed. From the repeated opportunities he has had of observing its action, Dr. P. has drawn the following corollaries:

1st. That this medicine acts in a particular manner upon the uterus, when that organ is the seat of an irritation which gives rise to active hemorrhage and when this bleeding results from chronic metritis.

2dly. In hemorrhage arising from acute metritis, it is necessary first to combat the inflammation by repeated sanguineous evacuations, and then to have recourse to the tannin.

3dly. It has no beneficial effect in those hemorrhages which are the result of organic alteration of the uterus.

4thly. This remedy ought to be preferred to all others in the treatment of uterine hemorrhage, not only on account of the promptitude with which it causes the symptoms to disappear, but because the dose necessary for this purpose is so small as not to disagree with the stomach even of debilitated and irritable persons.

---

*Oleum Ricini.*—M. LANGIER states that the repeated employment of this oil as a purgative, has twice produced on himself a pruriginous eruption, or redness and itching, at the wrists and bendings of the knees, yet the oil was neither rancid nor bitter.—*American Journal of Med. Sciences.*

---

*Abstract of a Paper entitled " Some Observations on the Effect produced by a Shock of Water in certain Morbid States of the System."* By CHARLES M. CLARKE, M.D. F.R.S. (Read at the College of Physicians.)

Dr. CLARKE in this paper alludes to the effect of a " sudden and lavish" application of water to the face, or the immersion of the whole body, in cases some of which have been generally ranked under the head of hysteria.

Such affections have generally occurred in females of a "pasty" complexion, fat, pale, and weak; or in such as have evinced the common signs of debility, such as feeble pulse, cold extremities, and purpleness of parts distant from the centre of circulation. The age of the patients has varied from ten to thirty years; in many, menstruation has been imperfect or absent, but without there being reason to regard this as the cause of the disease. The affection has consisted, 1, in actual loss of power of certain muscles, or an unwillingness to exert such power if it existed; 2, in an irregularity in certain muscular actions. These have apparently been brought on by indolence in some cases; in others, by over application of the mind; and, in yet others, by sudden and violent emotions. The muscles chiefly implicated have been those of the throat, neck, and lower extremities; but the author does not include chorea among cases of this nature, however beneficial cold bathing may be in that disease; nor are his remarks extended to any form of tetanus.

Sometimes the disease alluded to by Dr. Clarke appears in the form of spasmodic cough, which comes on " several times in every minute during the whole day" for many weeks, except when the patient is eating or sleeping. Sometimes the voice is suddenly lost; at others, the patient barks like a dog, or makes other " offensive and unintelligible noises." With regard to deglutition, sometimes the patient can swallow large morsels of food, or drink large quantities of fluid, but is unable to make the muscles act upon small quantities, and lets the saliva run from the mouth. In some examples the power of swallowing has been so entirely lost as to render it necessary to introduce food into the stomach through a tube. Sometimes the diaphragm is affected as in hiccup; in others, the breathing is very imperfectly performed, and the oxygenation of the blood interrupted. Lock jaw is uncommon, but an affection of the muscles of the neck, resembling opisthotonous, is not unfrequent.

When the muscles of the lower extremities are affected, the most common condition is a total loss of power, so that the thigh cannot be drawn up towards the body; or, if the limbs be bent by another person, the patient cannot straighten them again. In some cases Dr. Clarke has known this continue for many years, till at length the muscles have shrunk and the limbs lost their natural form. The arms are much less frequently affected, and scarcely ever are both together. If cold water will cure all or any of these — then —

The affection of the lower limbs, above described, bears no resemblance to paraplegia; there being no evidence of pressure on the brain or spinal column, no numbness of the surface, nor tingling of the limbs.

The absence of constitutional symptoms, and the performance of the natural functions, the author observed, had led many to suspect that such cases were feigned; but he does not think this probable, when it is considered that the cases so frequently agree with each other, without any possibility of this being the result of imitation, and that the patients are debarred the enjoyments of life, and their prospects blasted by the notoriety of their disorder. If it be argued that fear is a powerful agent in removing them, it may be answered that this is a very strong incentive to action under other circumstances, and frequently produces exertions which could not otherwise have been made.

There are certain circumstances which contraindicate the use of this remedy: viz. disease of the brain, lungs, or abdominal viscera, but particularly of the heart or great vessels. These, even if suspected, should prevent this treatment from being adopted. With regard to its application, it is generally sufficient to pour from two to four gallons of water (warm or cold, according to circumstances,) over the face, only allowing a short interval for the patient to breathe. The most convenient way is to place the patient with the head hanging over the edge of the bed, while she is firmly held by attendants. The first or second application is generally sufficient; but if, after two or three trials, it fails to give relief, it is not worth while to persevere. In old cases, and those which are severe, the whole body must be immersed in water several times, with "very short intervals" between the plunges. The patient not unfrequently suffers from severe headach afterwards; which, however, may be relieved by placing her in warm blankets, and exhibiting diffusible stimulants, as ammonia.

Four cases were mentioned in illustration. In one, the patient, about twenty-five years of age, was affected with very violent spasms of the diaphragm. A great variety of remedies had been employed without avail. She was cured by the cold affusion twice repeated. In another, a young lady aged twenty-two, there were spasms of the diaphragm, which impeded breathing, and the power of swallowing was lost, so that it was necessary to feed her through a tube. Menstruation was interrupted; the bowels sluggish; the patient could not stand. After many remedies had been tried, recourse was had to plunging her into cold water. She had severe headach, requiring ammonia. Her breathing was immediately relieved, and the power of deglutition restored in a slight degree. She recovered.

In a third instance, the patient was only nine years of age: she had spasmodic cough incessantly, except when she was eating or sleeping. The cough was instantly cured by cold affusion.

In a fourth patient, a young lady aged fourteen, a violent spasmodic affection of the diaphragm and larynx, which greatly interfered with deglutition, was cured with equal rapidity by similar means.—*Medical Gazette.*

### SURGERY.

*Removal of a Tumor from the right Nympha, by the Application of a Jugum.* By A. B. GRANVILLE, M.D. &c.

The following description of this very interesting case was lately submitted to the Westminster Medical Society by Dr. Granville. The instrument employed was also exhibited. We saw the patient a short time after the operation: her health was nearly reestablished, and the part operated upon was almost entirely healed.

The removal of pendulous tumors, or parts morbidly enlarged, connected with the structure of the external genitals in females, has long engaged my attention, in consequence of the frequent applications made at the Westminster General Dispensary by patients labouring under complaints of that description; and also from a considerable number of similar cases having occurred in my private practice within the last fourteen years. The methods usually employed for affording effectual relief in the majority of these cases, namely, excision and the application of ligatures, I have at times, in common with other practitioners, found to be attended with disadvantages, which have either deterred the patient or her attendant from resorting to them. I need hardly mention that I allude to the hemorrhage, sometimes formidable, which must necessarily follow in adopting the first method; and to the difficulty which will occasionally be encountered in accomplishing the second process. Indeed, the probability of serious hemorrhage succeeding the removal of all such tumors, or enlarged parts, when of considerable size, has induced, of late, many of the most eminent surgeons to abandon that practice nearly altogether, and to resort to the application of ligatures. If the connexion of the part to be removed with the healthy structure be by means of a round base, however large, one, two, or more ligatures may be applied without inconvenience; but if the connexion of the part to be removed be by means of a flat and lengthened attachment, the ligature, or ligatures, may not be so easily or effectually applied, inasmuch as there must be a puckering, or drawing together, of a considerable flat surface in a healthy structure below the disease, in order effectually to check the course of life in the latter; an operation attended by the most excruciating pain, and the almost inevitable risk of laceration.

These considerations induced me to devise a simple instrument, or *jugum*, by which, in all cases of tumors to be removed, having a flat and lengthened attachment, the effect of a single ligature is produced in an instant. The Society has an opportunity this evening of inspecting, in the preparation which lays before them, the result of the application of this instrument. The case is briefly this:

A young married woman consulted me a short time ago on account of some disease affecting the external organ of propagation. On inspection, I found that the right nympha, from the clitoris to the furca, measuring in length about two inches and three fourths, was morbidly enlarged, and to a considerable size, being partly scirrhous and partly vascular, and exhibiting several points of ulceration on its internal, and numerous points of abrasion on its external surface. The left nympha appeared affected by a similar disease, but in size was about one third only of the right. The local complaint had become a source of great constitutional irritation and disturbance; and there was considerable discharge both from the part itself and the vagina. The sufferings it produced were constant and increasing, and the poor woman begged earnestly to be relieved from her burthen. I ascertained from her that it had been treated as a syphilitic affection, although she was disposed to attribute the disease to a very difficult labour she had had the last time she was pregnant; but it also appeared from her report that none of the remedies employed had arrested the disorder. Under these circumstances I proposed to her the removal of the tumor; and, as soon as the jugum, of the size of the attachment of the tumor, could be got ready, it was applied, and the circulation between the latter and the healthy structure completely interrupted in less than half a minute.

Excision was out of the question in this case, for the vascularity of the part was excessive; and it would not have been a very easy matter to take up all the bleeding vessels. Besides, I could gain nothing in the way of sparing pain to the patient by having recourse to excision; and, as to time, I knew that in four days, or five days at the utmost, the removal of the tumor would be effected by pressure with the jugum. Such was, in fact, the result; and the preparation now on the table of the Society exhibits the tumor which came away at the end of the fifth day after the application of the instrument. Its vascularity shows that it would have been hazardous to have employed excision; while the great length of its attachment demonstrates that at least three ligatures must have been employed, in order to avoid laceration of the healthy skin; and that the application of three ligatures must be a complicated operation, compared to the mere application of an instrument which the patient herself might have easily adapted to the part. Moreover, even the mere insertion of a needle through two parts, at least, of the healthy skin, for the purpose of applying the said ligatures, trifling as it may seem to the practitioner, is not so to the sufferer. Another advantage of the jugum is, that the operator may at pleasure, and in a second, tighten or relax the pressure, according to the symptoms which it may have produced at first; which advantage is not to be despised when it is considered that the sudden tightening of a ligature has produced, at times, formidable symptoms: whenever these have followed the application of a common ligature, there is no other means of relaxing its firm grasp but by cutting the ligature altogether; and hence the necessity of a fresh application of it to the part, after the constitutional symptoms have subsided; but, in the case of the jugum, two or three retrograde turns of the screw are sufficient to relieve the untoward symptoms; after which, pressure is again easily obtained. In the case of the young woman in question, I twice gave way to her solicitations, and unscrewed the instrument so as to relieve her of pain; circumstances which, I have no doubt, protracted the period of separation of the tumor by one day at least. I feel perfectly satisfied, however, that it will be better, in all such cases, to strangulate the tumor at once, and drown the pain in laudanum during the first twenty-four hours; and not to unscrew the jugum except where really formidable symptoms seem to arise from the pressure.

In answer to a question from the President, Dr. Granville further stated that, on the coming away of the tumor, the jugum came away also, leaving a healthy surface behind, and without being followed by a single drop of blood. —*Med. Gazette.*

*Case of extensive Suppuration and Death, succeeding the Prick of a Pin; with Remarks.* By David Cunninghame.

A woman pricked her finger with a pin. She applied poultices, but continued to get worse till the third day, when she consulted Mr. Cunninghame. An incision was made down to the bone: this, after a time, discharged some thin unhealthy pus. The absorbents inflamed; the first phalanx of the finger felt "benumbed;" and the bone was found to be in "a semi-dissolved state." Another incision was made over the second phalanx. The parts sphacelated, the glands in the axilla swelled, and on the seventeenth day fluctuation was perceived over the superior angle of the scapula. Counter openings were made; stimulating injections, tonics, opiates, &c. were used without avail; and she died on the thirty-eighth day from the receipt of the injury, and eighteenth from the opening of the abscess.

Mr. Cunninghame is of opinion that irritation is chiefly communicated through the nerves, exciting an unhealthy action in the system partially, or in the system generally; and that the predisposition to this condition may be hereditary, or the result of disease or of mode of life, and that it may exist as well in the apparently robust as in the weak.—*Glasgow Med. Journal.*

---

*A Case of Tumor successfully extirpated from the Face.* By J. Hutchison, Esq.—Dhean Dhass, fakhir, ætat. thirty-two, thin, sallow, and emaciated, and but nervous and weak in mind, applied to me for assistance about the beginning of February 1826, in consequence of a large tumor, which was attached to about two thirds, or between that and one half of the upper lip, but inclining more to the left side, where it extended up on the side, so high as within an inch of the eyelid. From the insertion of the levator muscles, which appeared to have become stronger from use, he had considerable power in raising the tumor. Over it the hairs of the mustachoes were thinly scattered, and in general the integuments were adherent to the tumor underneath, except high on the left side of the face.

The tumor was irregular on the surface, and seemed composed of distinct nodules or lobes, from the size of a plum to that of a small potato; and to me it appeared to have increased by single additions of this sort, but I was unable to ascertain this point from the patient. There were numerous large tortuous veins over its surface, and several scabs and cicatrices were observable; while on the under part there was an ulceration, quite superficial however, from which exuded a plentiful watery discharge resembling saliva.

At the left angle of the mouth, the boundary of the tumor was abrupt, as if it had formed a cyst by the condensation of cellular membrane; and about half an inch of the lip there appeared quite sound, while on the right side it passed very imperceptibly into the sound parts. On examination, the gum underneath appeared pretty healthy; but three or four of the teeth underneath the tumor were loose.

The patient gave the following account of himself: That it commenced as a small boil or pimple, which afterwards burst, and was painful; that it daily increased for eight years to the present time, when he applied to me; that in cold or rainy weather it was very painful; and that it sometimes discharged such quantities of blood that he fainted, and that his health was greatly impaired by these discharges, which I supposed must have been the result of the sloughing process. He likewise mentioned that the tumor inconvenienced him much, and prevented his sleeping from its weight, and by obliging him always to recline in the same posture. It could be freely handled without giving much pain. He was very anxious to have it removed, but I was apprehensive that integuments sufficient could not be preserved.

However, on the 15th of February, with the assistance of one of the young officers of the regiment, I removed it, according to his request. It weighed two pounds, apothecaries' weight. On examining the wound, I observed at the upper part that a very small portion of diseased substance remained, which I transfixed with a hook, and removed. I had no watch by me. The patient lost about eight or ten ounces of blood, and about six or seven vessels were secured, although some of them required to be irritated before they would bleed, from the faintness of the patient. Both ends of the ligatures were now removed, as union by the first intention was my great object. The sides of the lip were approximated, and secured by two harelip pins, while one stitch of the interrupted suture secured the middle of the wound on the cheek. The wound was now dressed in the usual way. The wound in the lip healed by the first intention, while that in the cheek granulated from the bottom, and all was healed up in about twenty-five days, although a small fistulous opening from the mouth remained for some days after all the rest had healed. The mouth afterwards appeared rather small and a little to one side, and there was some puckering of the integuments of the cheek.

I detained him till the 28th of April, when the cicatrix continuing good, and his general health having much improved, I discharged him. He suffered a good deal for three or four days after the operation from fever and a cough, occasioned by the discharge running into his mouth. The tumor appears to me to be the common vascular sarcoma of Abernethy.—*Trans. of the Med. and Phys. Society of Calcutta.*

---

### MIDWIFERY.

*Six successive Hip Presentations in the same Individual.*—Madame Q., large and well made, of good constitution. Her first accouchement was long and difficult; the hips presented, and various manipulations were adopted by her attendant, which caused great pain; but at length the delivery took place spontaneously. She was put to bed a second time, and the labour was much easier, being speedily terminated, although the presentation was the same as before. On the third occasion, as soon as the pains came on, an accoucheur was sent for, who remained with her above ten hours, when the labour gradually ceased, and the delivery did not take place till five weeks after.

The physician who relates the case was sent for to the lady during her fourth pregnancy. He found the os uteri thick and hard, with a little tumor at the left side, about the size of a nut, and which felt like a hemorrhoid. The pains continued, but without effect, and after some hours entirely ceased. In a month afterwards she was delivered without difficulty, the hips still presenting. A fifth accouchement was attended with similar circumstances, false labour supervening about the eighth month, and delivery three weeks after.

On the 11th of December last, Madame Q. was seized with labour pains for the sixth time, but which again subsided till the 8th January; the hips presenting as in every one of the preceding instances.

These presentations are neither rare nor difficult at the Maternité in Paris:

360 were met with in 20,000 cases, and of these only thirty required the interference of art. But the case above detailed is so far remarkable, because though the woman was well formed, and the pregnancy presented nothing extraordinary, yet the position of the fœtus was always the one above mentioned.—*La Clinique.*

---

### CHEMISTRY.

*Nitrate of Silver as a Test for Vegetable and Animal Matter.*—Dr. DAVY states that nitrate of silver, dissolved in pure water, is not altered by the sun's rays. If the minutest quantity of vegetable or animal matter is present, the solution is discoloured; and with common distilled water, the discoloration is strong. To prove that the cause of the change of colour is the one assigned, it is sufficient to allow the coloured matter to subside, decant the colourless solution, and expose it again to sunshine. However powerful the sun's rays are, no further effect is produced; but, add more common distilled water, and the phenomenon will instantly reappear. He believes nitrate of silver, thus used, is one of the best tests of the presence in water of very minute portions of vegetable matter: of course, any chloride of silver that may be formed in consequence of the presence of any muriates should be allowed to subside in the dark, and the subsidence should be complete before the fluid is decanted and exposed to light.—*Jameson's Journal.*

---

*On the Presence of Iron in Tin.*—From M. FISCHER's experiments it would appear, that even the very best tin contains small quantities of iron occasionally, which, entering into the compounds afterwards formed by the metal, cannot be easily separated. The best method to decide upon its presence is to decompose a salt formed from the tin by ammonia, and separate the protoxide which is thrown down. This precipitate is then to be digested in cold muriatic acid; nearly all the oxide dissolves; but, if the portion which last remains be dissolved, it will be found to contain but little oxide of tin, mixed with a large proportion of oxide of iron. This portion, acted upon by warm and strong muriatic acid, will dissolve; and then the iron may be recognised in the usual manner.—KASTNER, *Bull. Univ.* A. x. 313. (*Quarterly Journ. of Science.*)

---

### MISCELLANEOUS.

*Effects of Violence on the Body after Death.**—In the very interesting paper from which we select the following extract, Dr. CHRISTISON relates many experiments in which external violence was inflicted upon the body after death, for the purpose of instituting a comparison between the effects which follow in such cases, and those which result from injury before death.

In respect to external contusions, the experiments show that for some hours after death blows will cause appearances which, in point of colour, do not differ from the effects of blows inflicted recently before death; that the discoloration generally arises, like lividity, from an effusion of the thinnest possible layer of the fluid part of the blood on the outer surface of the true skin, but sometimes also from an effusion of thin blood into a perceptible

* From Dr. CHRISTISON's "Observations on Medical Jurisprudence." *Edinburgh Med. Journal,* April.

stratum of the true skin itself; and that dark fluid blood may be even effused into the subcutaneous cellular tissue in the seat of the discolorations, so as to blacken or redden the membranous partitions of the adipose cells; but that this last effusion is never extensive.

It can hardly be doubted that the appearances now described will exactly imitate slight contusions inflicted during life. But I conceive that the blows in the latter case must be trivial.

When a blow inflicted during life is more severe, it may have the following effects, few or none of which, so far as we know, can originate in violence after death: 1. There may be swelling from the extent of the extravasation. This is certainly never caused in the dead body. 2. When the violence has been applied a few days before death, there will be a yellow margin round the black mark, which is another appearance that cannot be formed except during life. 3. There may be clots of blood in the subjacent cellular tissue, either with or without swelling. This appearance I have never seen accompanying contusions caused in the dead body; but it may be doubted whether clots might not be formed, if the injury was inflicted very soon after death, and had the effect of lacerating a considerable vessel in the neighbourhood of loose cellular tissue. 4. In the instances in which the blood does not coagulate at all after death, contusions caused during life may be recognised by the extent of the effusion into the cellular tissue. In a part not liable to be infiltered by its depending position, and not in the vicinity of a large vein, a deep effusion of fluid blood, which fills and distends the cells of the cellular tissue, can hardly be produced on the dead body. 5. Perhaps one of the most characteristic signs of a contusion inflicted during life is incorporation of blood with the whole thickness of the true skin, rendering it black instead of white, and increasing its firmness and resistance. This sign may not be always present; for, as every one knows, a blow may cause extensive extravasation below the skin, without affecting the skin itself. But, when present, I am disposed to consider it characteristic, because I have never been able to produce it in the dead body, and it is not easy to conceive how such a change can be wrought in so dense a texture as the skin, without the force and agency of living vessels.

It is impossible to fix absolutely the limit of the interval beyond which contusions cannot be imitated by violence applied to the dead body. It appears to vary with the state of the blood and the time which elapses before the body cools and the joints stiffen. Sometimes the appearance of contusions can hardly be produced two hours after death; sometimes they may be slightly caused three hours and a quarter after it; but I should be inclined to think this period very near the extreme limit. Wherever the warmth of the body and laxity of the muscles were not considerable at the time the injury was inflicted, we may be sure that the appearance of contusions cannot be considerable. It is probably, therefore, only on the trunk that, even in the most favorable state of the body, namely, when the blood remains altogether fluid, any material mark of contusion can be produced so late as two hours after death.

As to *internal hemorrhage*, it is plain that if, in the dead body, a considerable blood-vessel, and more especially a vein, be lacerated so as to open into an extensive cavity or shut sac, there will be more or less effusion of the fluid part of the blood into the cavity. And even if the aperture in the vessel communicate only with the cellular tissue, percolation will take place to a notable extent, particularly when the level of the part is low in relation to the rest of the body.*

The hemorrhage and percolation will be peculiarly distinct in the cases in which the blood does not coagulate at all after death; for it seems then to acquire even a greater degree of fluidity than it possesses during life. We must not suppose that extravasations of blood within the body are not vital, merely because the effused blood is found fluid. Although vital effusions are usually coagulated, they are not so always; and, in particular, they are often fluid in the spinal canal. Professor BERNDT has mentioned such a case, the effusion having been caused by fracture of the cervical vertebræ;† M. OLLIVIER met with another, in which the effusion was caused by a wound of the middle meningeal vein with a small sword;‡ and Mr. CHEVALIER relates another, in which the hemorrhage was spontaneous.§ In all of them the blood effused into the spine was fluid, and in Bernt's case it was fluid every where. A circumstance worthy of mention here is, that the blood may continue permanently fluid in some parts or organs, while it coagulates as usual throughout the body generally, or perhaps in the heart alone. In the subject of one experiment it was coagulated in the heart, but fluid in the subclavian and spinal veins; in another it was firmly coagulated in the great veins of the abdomen, but quite fluid in the vessels of the spinal canal. The late Dr. MERTZDORFF, of Berlin, in a paper on the Effects of Blows after Death, has taken notice of this diversity in the appearance of the blood, and says it commonly appeared to him that the blood of the vessels within the head and spine, in the subclavian veins, and in the vena portæ, was fluid, even when it was coagulated in the other vessels. I have often had occasion to make the same remark. The inference to be drawn from the fact is, that the inspector must not hastily assume extravasations of fluid blood in these parts as having taken place after death, because he finds the blood coagulated in the heart and subordinate vessels, but must examine the state of the blood in the vessels adjoining the extravasation.

It may not always be easy to distinguish internal hemorrhage according as it occurs before or after death. Neither can I pretend at present to examine the subject in all its bearings. If any of the organs in the cavity bear marks of compression by the effused blood, the effusion must have been vital. So, likewise, if the cavity into which the hemorrhage has taken place be filled with blood, or if any of the softer viscera be comminuted, or broken down, or injected, by the blood bursting through their texture; or if the hemorrhage be considerable in relation to the size of the vessel, or have evidently proceeded from an artery, and be extensive in proportion to its size. If the effused blood be coagulated, and the coagulum not broken down, it must

* Dr. Christison has previously remarked in his paper, that "blood drawn from the jugular and femoral veins, eight hours after death, flowed out quite fluid, and in a few minutes formed a firm coagulum, with separation of serum. The clot was firm enough to bear tossing from hand to hand without breaking. Blood drawn from the femoral vein an hour and a half later, and which was losing its fluidity, formed, on standing, a thick diffluent mass, with separation of serum, but without a proper clot."

† Beiträge zur gerichtl. Arzneik. ii. 231.

‡ De la Moelle épinière, p. 254.

§ London Med. Chirurg. Transactions, iii.

|| Horn's Archiv für Mediz. Erfahrung, 1823, i. 280.

have taken place either before death or very soon after it. A state of the blood, the reverse of that mentioned under each of the foregoing propositions, will render the date of the hemorrhage at all events equivocal. A small, or even moderate, effusion from the rupture of an artery of considerable size could hardly have occurred during life. An effusion of fluid blood from vessels in the neighbourhood of which it is coagulated, must have occurred in the dead body. The most doubtful appearance of all is, when the effusion is fluid, moderate in quantity, unaccompanied by the rupture of any considerable vessel, but connected with fluidity of the blood throughout the body, or in the vessels near the cavity into which the hemorrhage has taken place.

The interval after death within which vital hemorrhage into the internal cavities may be imitated by violence to the dead body, will vary with the qualities of the blood. When the blood has not lost its power of coagulating in the body, the violence must be applied before it coagulates; which appears to happen soon after the stiffening of the muscles begins. When it continues altogether fluid, there seems no limit to the time at which imitative hemorrhage may be produced, except great decay of the body. In Experiment 3, as well as in the body of the woman Campbell, it was produced about eighteen hours after death. At this period all the changes must have occurred which the body undergoes prior to putrefaction; and, when putrefaction has begun, imitative hemorrhage may be caused still more readily, nay, without the cooperation of external violence.

---

*Silver detected in the internal Viscera.*—Dr. Wedemeyer, of Hanover, has recorded the case of a person who had used the nitrate of silver internally for eighteen months, for the cure of epilepsy. The cure was effected, and the skin became discoloured. The patient was attacked with hepatic disease and ascites, of which he died. Upon dissection, all the internal viscera were found more or less stained of a blue colour, in the same manner as the external surface. The plexus choroides and pancreas were submitted to examination by M. Brande, and a portion of metallic silver obtained.—*Rust's Repert.*

---

### NATURAL HISTORY.

*Curious Hybrid.*—There is now at Berlin, an animal produced between a stag and a mare. The appearance of the creature is remarkable; the forepart is that of a horse, the hind part that of a stag, but all the feet are like those of the latter animal. The king has purchased this hybrid, and sent it to the menagerie at Potsdam.

---

*Observations on the Mantis Tribe, or that of the Leaf Insects,* by Dr. Adam. Read at a meeting of the Physical Committee of the Asiatic Society of Calcutta.

Of all the insect tribes in India, that of the Leaf Insects is the most remarkable for external form. According to the latest classification, this tribe has been divided into the families of the Mantida and Phasmida, founded on a difference in the structure of the foot and leg; this member in the former being raptorious, is provided with a sharp claw, and a hollow on the leg and thigh, and a double series of spurs, for the better securing its prey; and in the latter being destitute of any such peculiarity. Dr. Adam calls two of the specimens laid before the Committee *Gongylodes*, as they appear to correspond closely with the description and figure of that species in the latest entomological works. This insect, when alive and fresh, presents a striking resemblance to a blade of grass, differing in colour according to the season, being green and succulent in the rains, and in the dry weather so much like a withered straw, that they can with difficulty be distinguished. On first beholding this insect, during the hot winds, in the upper provinces, Dr. Adam could hardly be convinced that it was not a straw, and part of the same long and dry grass on which it rested. A slight movement of the head, however, like that of the house-lizard on the wall, when watching its prey, satisfied him that it was a living object, and on removing grass and all to his hut for examination, he was both surprised and amused at the extraordinary powers which the insect developed. Clinging close to the upright straw, which was fixed on the table, the animal lay in wait for its prey with no less design than would be exhibited by a cat, or tiger, and if an unlucky fly happened to alight in his neighbourhood, there was hardly left to it a chance of escape. He projects rapidly his armed paw, and with unerring aim transfixing his victim, lodges it in the toothed hollow of the thigh, destined for its reception. After the fly is in his power, no time is lost in devouring it, commencing with the trunk, and in a few minutes swallowing the whole, the head and wings constituting the finishing morsel. In this manner he would destroy at a meal five or six large flies, which in point of bulk nearly doubled his own body. On viewing the structure of the fore-limb of this insect, it seems impossible to imagine any thing more perfectly contrived for the end in view. The limb itself so strong and muscular, provided with a claw at its extremity, likewise strong, horny, and sharp as a needle; and the groove in the last joints, with the double row of teeth or spurs on the margin, corresponding and locking closely into one another, like the fangs of the alligator, altogether constitute an apparatus for seizing and securing its prey, which in so small a creature cannot but excite admiration. By means of these formidable weapons, the insect not only becomes destructive to others, but is employed to attack its own species; and in China, we are told, fighting the Mantis forms as much the favorite amusement of boys, who carry them about in cages for that purpose, as cock-fighting in England, or among the inhabitants of the Eastern Islands. —*Edinburgh Journal of Science.*

